# 166 Identifying Resilient Systems in Outpatient Hemodialysis Using Machine Learning

**DOI:** 10.1017/ash.2026.10567

**Published:** 2026-06-23

**Authors:** Donna Nucci, Erica Plummer, Oliver Mayorga, Jaspreet Benwait

**Affiliations:** 1 Yale New Haven Health; 2 Yale New Haven Health System

## Abstract

**Background** Central-line–associated bloodstream infections (CLABSIs) are healthcare-associated infections occurring in critically-ill patients where central veins are accessed using central venous catheters (CVCs). Use of antimicrobial CVCs has been recommended to reduce CLABSI rates in acute care settings. 1 Reducing CLABSI rates in community hospitals remains a challenge due to variability in the type of CVC used, CVC bundle compliance, interdisciplinary collaboration, and overall culture related to central line (CL) use. Methods A quality improvement project (QIP) conducted at two acute care community hospitals evaluated the impact of standardized CL insertion bundle, interdisciplinary collaboration, and shared accountability on CLABSI rates. The QIP was conducted at facilities, A and B, within the same hospital system, from October 2018–September 2025, where both facilities used a CVC kit containing a silver-platinum-carbon (SPC)-impregnated CVC for vascular access. From January 2020–June 2024, in addition to the CL kit, key interventions implemented sequentially included daily infection prevention (IP) emails regarding CL status; multidisciplinary CLABSI rounds to discuss CL indications/culturing practices; chlorhexidine gluconate (CHG) bathing documentation transition to medical administration records; optimization of CVC device maintenance order placement in an electronic health record system; initiation of CHG/isopropyl alcohol device swab for hub scrubbing for all CL access; and blood cultures ordered through a care pathway incorporating blood culturing stewardship. CLABSI rates were calculated in terms of events per 1,000 CL days to assess the impact of these interventions on CLABSI prevention. Results The post-intervention CLABSI rate for Facility A was significantly lower than the pre-intervention rate (0.100 vs 0.728; P = 0.0409). The post- and pre-intervention CLABSI rates for Facility B were 0.494 and 0, respectively; although clinically significant, no statistical difference in rates was observed (P = 1). For both facilities, the observed monthly CLABSI rates exhibited a gradual long-term decline without significant impact of specific interventions. The decline was statistically significant for Facility A (P = 0.00992) whereas for Facility B the decline was non-significant (P = 0.152). Conclusions Continuous implementation of the SPC-impregnated CVC-containing kit likely established a stable baseline for infection prevention. Subsequent interventions did not result in statistically significant changes in monthly CLABSI rates, suggesting gradual improvements attributable to ongoing process maturation rather than discrete intervention effects. References

